# Pathogenic Potential to Humans of Bovine *Escherichia coli* O26, Scotland

**DOI:** 10.3201/eid1803.111236

**Published:** 2012-03

**Authors:** Margo E. Chase-Topping, Tracy Rosser, Lesley J. Allison, Emily Courcier, Judith Evans, Iain J. McKendrick, Michael C. Pearce, Ian Handel, Alfredo Caprioli, Helge Karch, Mary F. Hanson, Kevin G.J. Pollock, Mary E. Locking, Mark E.J. Woolhouse, Louise Matthews, J. Chris Low, David L. Gally

**Affiliations:** University of Edinburgh, Edinburgh, UK (M.E. Chase-Topping, E. Courcier, M.C. Pearce, M.E.J. Woolhouse);; The Roslin Institute and Royal (Dick) School of Veterinary Studies, Edinburgh (T. Rosser, I. Handel, D.L. Gally);; Scottish *E. coli* O157/VTEC Reference Laboratory, Edinburgh (L.J. Allison, M.F. Hanson);; Scottish Agricultural College, Edinburgh (J. Evans, M.C. Pearce, J.C. Low);; Biomathematics and Statistics Scotland, Edinburgh (I.J. McKendrick);; Istituto Superiore di Sanità, Rome, Italy (A. Caprioli);; University of Münster, Münster, Germany (H. Karch);; Health Protection Scotland, Glasgow, UK (K.G.J. Pollock, M.E. Locking);; University of Glasgow Veterinary School, Glasgow (L. Matthews)

**Keywords:** *Escherichia coli* O26, *Escherichia coli* O157, prevalence, pulsed-field gel electrophoresis, PFGE, multilocus sequence typing, MLST, nonmetric multidimensional scaling, NMS, bacteria, Scotland

## Abstract

*Escherichia coli* O26 and O157 have similar overall prevalences in cattle in Scotland, but in humans, Shiga toxin–producing *E. coli* O26 infections are fewer and clinically less severe than *E. coli* O157 infections. To investigate this discrepancy, we genotyped *E. coli* O26 isolates from cattle and humans in Scotland and continental Europe. The genetic background of some strains from Scotland was closely related to that of strains causing severe infections in Europe. Nonmetric multidimensional scaling found an association between hemolytic uremic syndrome (HUS) and multilocus sequence type 21 strains and confirmed the role of *stx_2_* in severe human disease. Although the prevalences of *E. coli* O26 and O157 on cattle farms in Scotland are equivalent, prevalence of more virulent strains is low, reducing human infection risk. However, new data on *E. coli* O26–associated HUS in humans highlight the need for surveillance of non-O157 enterohemorrhagic *E. coli* and for understanding *stx_2_* phage acquisition.

## Abstract 2

This pathogen may be the next Shiga toxin–producing *E. coli* of concern.

Enteropathogenic *Escherichia coli* (EPEC) and enterohemorrhagic *E. coli* (EHEC) are gastrointestinal pathogens associated with asymptomatic carriage and human diseases ranging from mild diarrhea to hemorrhagic colitis and hemolytic uremic syndrome (HUS) (*1*). Worldwide, EHEC serogroup O157 strains are responsible for most human Shiga toxin–producing *E. coli* (STEC) infections (*2*). However, recent outbreaks in Germany (*3*) and the United States (*4*) have highlighted the increasing role of other Shiga toxin–producing serogroups (termed non-O157 strains) in causing human disease.

The pathogenesis of EHEC strains is associated with production of Shiga toxins expressed from lysogenic bacteriophages in the EHEC genome. There are 2 predominant classes of Shiga toxins (1 and 2), each encoded from 2 genes, *stxAB*, with the genotypes simplified to *stx_1_* or *stx_2_* in this study. Whereas EHEC is regarded as an emerging zoonotic pathogen, the related EPEC strains cause diarrhea, especially in infants in developing countries (*1*).

EPEC and EHEC express a type III secretion (T3S) system that translocates multiple effector proteins into host cells and manipulate host innate responses, which are needed for colonization (*5**–**7*). The T3S system is central to the formation of attaching and effacing lesions on the intestinal epithelium that requires the bacterial outer membrane protein intimin (encoded by *eae*) and the secreted bacterial protein—the translocated intimin receptor (Tir)—that is injected into the host cell. Studies of EPEC and EHEC O157 show that these pathogens trigger different actin polymerization pathways respectively involving Tir cytoskeleton coupling proteins (*tccP* or *tccP2*) (*5*). The formation of attaching and effacing lesions is needed for bacterial colonization by EHEC O157 and EHEC O26 in cattle. However, Shiga toxin (Stx) is the principal factor responsible for severe human illness, including HUS (*8*). Strains with *stx_2_* alone appear more strongly associated with HUS than do strains with only *stx_1_* (*8**–**10*). These observations have been supported by mouse (*11*) and primate models (*12*).

Among non-O157 EHEC, Stx-producing *E. coli* O26 also causes human disease (*13**–**15*) and has been isolated from livestock (*16*). However, unlike EHEC serogroup O157, it may be pathogenic for both cattle and humans (*17*). Although the origin of human *E. coli* O26 infections is rarely identified (*18*), evidence exists of person-to-person spread (*19*) and foodborne transmission (*20*). Furthermore, because EHEC O26 has been isolated from the feces of cattle and other animals (*16**,**21**,**22*), potential exists for direct and indirect zoonotic transmission to humans (*20*).

In the United Kingdom, Stx-producing human *E. coli* O26 infections are usually uncommon and not clinically severe. Most identified EHEC infections are associated with serogroup O157, and incidence rates in Scotland are among the highest, compared with rates in countries with comparable surveillance (*23*). In contrast with human infection rates, farm and animal prevalence of *E. coli* serogroups O26 and O157 are similar in Scotland (*21*).

Our first objective was to identify the cause of the disparity between the incidence of *E. coli* O157 and O26 infections among humans in Scotland by examining the natural heterogeneity among serogroup O26 strains and reexamining their prevalence in cattle. As a second objective, we used different molecular techniques to compare the relationships between *E. coli* O26 isolates recovered from humans and cattle.

## Methods

### Bacterial Isolates

All bacterial isolates used in this research are shown in Table 1. All isolates from cattle were collected in parallel with a prevalence study of *E. coli* O157 in cattle in Scotland, conducted during 2002–2004 (*21*). Fecal samples were obtained from cattle on 338 farms to test for non-O157 *E. coli* strains, including serogroup O26, as described by Pearce et al. (*21*). Human *E. coli* O26 isolates came from collections within the United Kingdom, Ireland, and continental Europe.

**Table 1:** Source of *Escherichia coli* O26 isolates and sample sizes for the various statistical analyses, Scotland*

Source	Country of origin	Analyses	No. isolates	Clinical information
Bovine†	Scotland	Prevalence	249	NR
Bovine†	Scotland	PFGE	154	NR
Bovine†	Scotland	MLST	33	NR
Human‡	Scotland	PFGE	12	NK
Human‡	Scotland	MLST	11	D (n = 8); BD (n = 2); NK (n = 1)
Human§	England	PFGE	4	NK
Human§	Ireland	PFGE	11	NK
Human§	Belgium	PFGE	2	NK
Human§	Sweden	PFGE	3	NK
Human§	Italy	PFGE	1	NK
Human¶	Italy	MLST	5	HC (n = 1); HUS (n = 4)
Human#	Germany	MLST	14	D (n = 6); HUS (n = 8)

*NR, not relevant; PFGE, pulsed-field gel electrophoresis; MLST, multilocus sequence typing; NK, not known; D, diarrhea; BD, bloody diarrhea, HC, hemorragic colitis; HUS, hemolytic uremic syndrome. Where appropriate, the number of isolates from each clinical designation is provided. †Isolated 2002–2004 (*21*). ‡L.J. Allison, Scottish *E. coli* O157/VTEC Reference Laboratory, Edinburgh, UK; isolated 2002–2003. §H. Smith, Laboratory of Gastrointestinal Pathogens, Colindale, UK. The years isolated are unknown. ¶A. Caprioli, Instituto Superiore di Sanità, Rome, Italy. The years isolated are unknown. #H. Karch, University of Műnster, Műnster, Germany; isolated 1994–2000.

### PCR for Virulence Genes

Genes encoding *stx_1_*, *stx_2_*, *eae*, and *hlyA* were detected by using multiplex PCR (*24*). Possession of the locus of enterocyte effacement (LEE) pathogenicity island was confirmed by PCR screening for *sepL*. Genes encoding *tccP* and *tccP2* were detected with PCR by using universal primer pair univtccP/tccP2-F and tccP-R (*25*). Primers and PCR conditions used in this study are listed in Table 2.

**Table 2:** Primers and PCR conditions for *Escherichia coli* O26, Scotland

Primer name	Primer sequence, 5′ → 3′	Target gene	Annealing	Amplicon size, bp	Reference
stx1F stx1R	ATAAATCGCCATTCGTTGACTAC AGAACGCCCACTGAGATCATC	*stx_1_*	60°C,45 s	180	(*24*)
stx2F stx2R	GGCACTGTCTGAAACTGCTCC TCGCCAGTTATCTGACATTCTG	*stx_2_*	60°C,45 s	255	(*24*)
eaeAF eaeAR	GACCCGGCACAAGCATAAGC CCACCTGCAGCAACAAGAGG	*eae*	60°C,45 s	384	(*24*)
hlyAF hlyAR	GCATCATCAAGCGTACGTTCC AATGAGCCAAGCTGGTTAAGCT	*hlyA*	60°C,45 s	534	(*24*)
sepLF sepLR	GCTAAGCCTGGGATATCGC ACAATCGATACCCGAGAAGG	*sepL*	60°C,45 s	725	This study
univ tccP/tccp2-F tccP-R	GTAAAAACCAGCTCACCTTTTTC TCACGAGCGCTTAGATGTATTAAT	*tccp* *tccP2*	64°C,60 s	Variable	(*25*)
espAF espAR	CCTTCTCGGGTATCGATTGTCG CAGAGGGCGTCACTAATGAGTG	*espA*	58°C,60 s	1012	This study
LEE1promF LEE1promR	CGAATGGTACGGTTATGCGGG GCTCTCGCAGTCGCTTTGCTTCC	*LEE1*	58°C,60 s	645	This study

### Pulsed-field Gel Electrophoresis

We performed pulsed-field gel electrophoresis (PFGE) on 187 isolates (33 from humans and 154 from cattle) (Table 1) by using the method of Willshaw et al. (*26*), standardized by the Scottish *E. coli* O157/VTEC Reference Laboratory (SERL; Edinburgh, UK) and the Laboratory of Gastrointestinal Pathogens (Colindale, UK). A phage λ ladder (48.5 kb) was used as a DNA size standard, and gels were run with a linearly ramped switch time of 5–50 s applied for 38 h at a voltage of 5.4 V/cm and an included angle of 120°. PFGE profiles were analyzed by using BioNumerics (version 3.0) software (Applied Maths, Kortrijk, Belgium). The degree of similarity between profiles was determined by the Jaccard coefficient, and dendrograms were generated by using the unweighted pair group method with arithmetic mean with 1.3% tolerance and 1% optimization settings.

### Multilocus Sequence Typing

We performed multilocus sequence typing (MLST) on 63 isolates (30 from humans and 33 from cattle) (Table 1) by using a method similar to that of Wirth et al. (*27*). Internal fragments of 7 housekeeping genes (*adk*, *fumC*, *gyrB*, *icd*, *mdh*, *purA*, and *recA*) were sequenced, and the allele numbers and sequence types (STs) were assigned in accordance with the *E. coli* MLST database (http://mlst.ucc.ie/mlst/dbs/Ecoli). The T3S system of *E. coli* O26 is encoded on the LEE, and this region was originally sequenced from *E. coli* O26 isolate 413/89–1 (GenBank accession no. AJ277443). From this sequence, primers were designed to amplify *espA* encoding the main translocation filament protein of the T3S system and a 644-bp region (5082–5726) including the promoter controlling expression of the first LEE operon (*LEE1*) (Table 2). Sequence data for *espA* and the *LEE1* promoter region were compared by using ClustalX (www.clustal.org). Each unique sequence was given a different allele number assigned in the order in which they were discovered.

### Data Management and Statistical Analyses

#### Prevalence of *E. coli* O26

**Figure 1:**
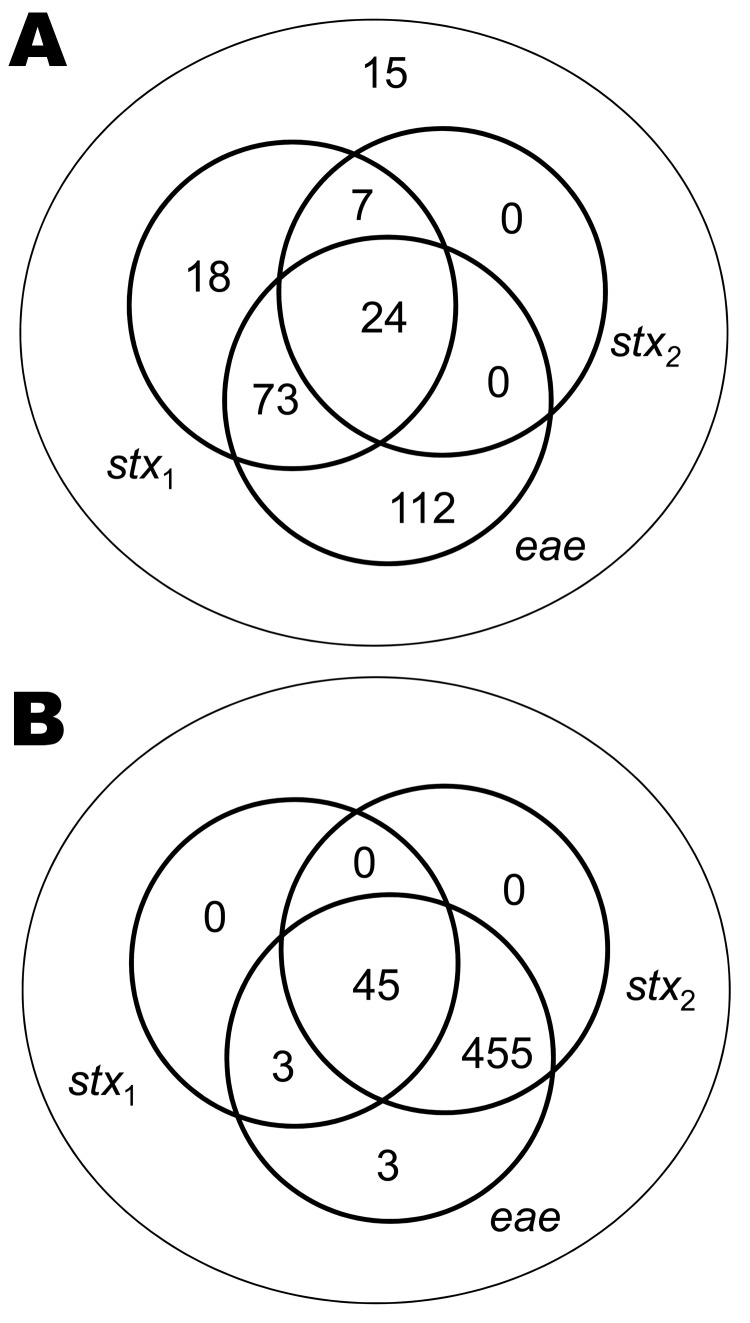
Isolates of *Escherichia coli* O26 (A; n = 249) and O157 (B; n = 507), collected from a 2002–2004 field survey, that illustrate the differences between the 2 serogroups from Scotland with reference to the presence or absence of Shiga toxin gene (*stx_1_* and *stx_2_*) and the *eae* gene.

*E. coli* O26 isolated from cattle are more heterogeneous than *E. coli* O157 with respect to the presence of *stx* and *eae* (Figure 1). Therefore, we determined the prevalence of *E. coli* O26 for 4 different groups with potential differences in virulence: 1) all *E. coli* O26, similar to Pearce et al. (*21*); 2) *stx_1_*+; 3) *stx_1_*+*stx_2_*+; and 4) *stx_2_*+*eae*+. Farm-level and fecal pat–level prevalences were calculated by using the method of Pearce et al. (*28*). SAS version 9.1.3 (SAS Institute, Cary, NC, USA) was used to fit generalized linear mixed models and to generate bootstrap-based estimates of key parameters. Excel 2000 (Microsoft Corporation, Redmond, WA, USA) was used to implement a Latin hypercube sampling to convert results from generalized linear mixed models into prevalence taking into account random effects (*29*).

#### PFGE Analysis

Differences between isolates from humans and cattle were evaluated by assigning isolates to statistically distinct groups. The methods used are described in (*30*). Group membership was determined by estimating an optimum cutoff value using dendrogram-based distances. Statistical support for each group was evaluated by using the cophenetic correlation. The statistical significance of the cophenetic correlation was evaluated by using a Mantel test. Program zt (*31*) was used with 1,000 simulations, and the probability of the observed cophenetic correlation occurring by chance was <5% for groups of isolates regarded as statistically distinct.

A subset of 41 isolates from Scotland (8 from humans and 33 from cattle) were included in the MLST and PFGE analyses. The PFGE data for 3 isolates from humans were missing and therefore could not be included in the comparison. Agreement between these 2 techniques was examined.

#### Nonmetric Multidimensional Scaling

To identify patterns in data associated with the clinical severity of infection in humans, we used nonmetric multidimensional scaling (NMS) to analyze all genotype characteristics measured. PC-ORD software version 6.03 (MjM Software Design, Gleneden Beach, OR, USA) was used. The main matrix was created from the following isolate data: MLST type (ST, ST complex), *tccP* and *tccP2*, Stx status, *hlyA*, *eae*, LEE, and sequence upstream region of *LEE1*. A second matrix that used isolate data and an indicator of clinical severity was also created. Isolates were separated into 2 groups: 1) HUS and those associated with HUS and 2) non-HUS, including those from diarrhea, bloody diarrhea, and hemorrhagic colitis. The grouping was chosen because HUS has clearly identifiable clinical characteristics and is a reliable indicator of severe illness.

We used NMS with a Euclidian distance measure after standardizing each variable by division by its standard deviation. The dimensionality of the dataset was determined by plotting an inverse measure of fit (“stress”) to the number of dimensions. Optimal dimensionality was based on the number of dimensions with the lowest stress. A 3- dimensional solution was shown to be optimal. We performed several NMS runs to ensure that the solution was stable and probably represented a configuration with the best possible fit. For each NMS run, we used 500 iterations with random starting coordinates. The overall significance of the difference between HUS and non-HUS groups was assessed by using multiresponse permutation procedures (*32*).

## Results

### Prevalence in Cattle

**Figure 2:**
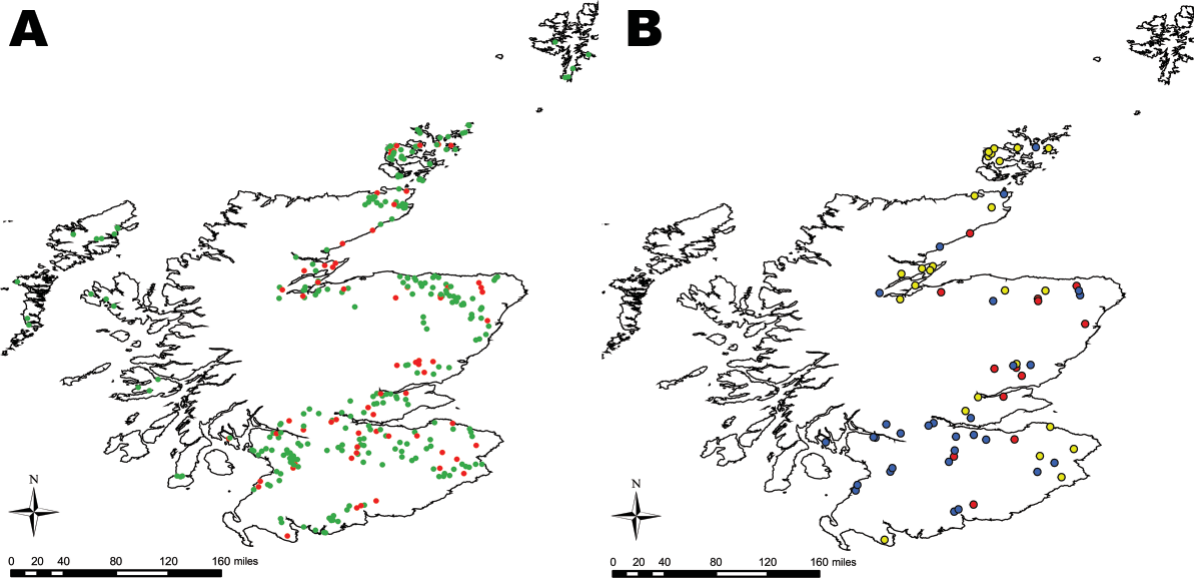
Location of farms sampled in 2002–2004 field survey for *Escherichia coli* O26, Scotland. A) Farms that were positive for *E. coli* O26 are shown in red; farms negative for *E. coli* O26 are shown in green. B) The positive farms were subdivided according to differences in virulent properties of *E. coli* O26 (farm status) based on the possession of stx. Farms were designated as *stx–* (blue), *stx_1_*+ (yellow), or *stx_1_*+*stx_2_*+ (red).

Of the 338 farms visited, 68 were positive for *E. coli* O26 (mean prevalence 20.1%, exact binomial 95% CI 16.0–24.8); these 68 farms were evenly distributed across Scotland (Figure 2, panel A). Farms were classified by their isolates’ virulence groups: *stx*–, *stx_1_*+, and *stx_1_*+*stx_2_*+. No farm had *stx_2_*+ only strains. Farms with *stx*– isolates clustered in southern Scotland. Farms that were *stx_1_*+ were fairly evenly distributed, but those with *stx_1_*+*stx_2_*+ isolates were found mainly in the northeast (Figure 2, panel B). Analysis of the farms grouped according to their Scottish animal health district (*21*) significantly supported this observation (p = 0.005). No seasonal differences were observed (p = 0.59).

The adjusted mean overall prevalence of *E. coli* O26 was 0.22 (95% CI 0.18–0.27) and 0.046 (95% CI 0.031–0.062), respectively (Table 3). However, the adjusted mean prevalences for farms and fecal pats of *stx_2_*+*eae*+ *E. coli* O26 were lower at 0.05 (0.03–0.09) and 0.004 (0.001–0.007), respectively.

**Table 3:** Farm-level and fecal pat–level prevalence of *Escherichia coli* O26, Scotland*

*E. coli* O26 status	Farms, n = 338				Fecal pats, n = 6,086		
	No. positive	Observed prevalence	Adjusted prevalence (95% CI)		No. positive	Observed prevalence	Adjusted prevalence (95% CI)
*E. coli* O26*	68	0.20	0.22 (0.18–0.27)		249	0.041	0.046 (0.031–0.062)
*stx*+ *E. coli* O26	38	0.11	0.12 (0.09–0.16)		122	0.020	0.020 (0.012–0.029)
*stx_1_* + *stx_2_* + *E. coli* O26	13	0.04	0.06 (0.031–0.09)		97	0.016	0.004 (0.001–0.008)
*stx_1_* + *stx_2_* + *eae* + *E. coli* O26	12	0.04	0.05 (0.03–0.09)		24	0.004	0.004 (0.001–0.007)

*Minor differences in the farm-level prevalence estimates for *E. coli* O26 between Pearce et al. (*21*) and this study resulted from use of different statistical models. Pearce et al. (*21*) aimed to provide national prevalence estimates; thus, weighted estimates of mean prevalence were generated that accounted for the fractions of the national herd found in different Animal Health Districts (AHDs). By contrast, we reported the mean of the sample collected in a stratified fashion across the AHDs. Because there were only relatively small differences in mean prevalence in the different AHDs, the effect on the 2 means is negligible and the effect on the standard errors relatively small.

### PFGE Genotyping

PFGE profiles of the 187 *E. coli* O26 isolates from 154 cattle and 33 humans were compared (Technical Appendix Figure 1). This comparison identified 4 groups comprising 5, 19, 142, and 16 isolates, which all included *E. coli* O26 isolates from cattle and humans. The 4 groups are characterized by statistically significant cophenetic correlations ranging from 0.807 to 0.945. Isolates from groups 2 and 4 were mainly *stx*–, whereas groups 1 and 3 were mainly *stx*+. Five isolates were not assigned to a group. All of the *stx*+ isolates from outside Scotland were in group 2, along with the 5 *stx*+ isolates from Scotland. The remaining isolates from Scotland were in group 1 (2 isolates), 2 (3 isolates), and 4 (1 isolates).

We compared PFGE profiles of the 41 *E. coli* O26 isolates from 33 cattle and 8 humans. PFGE grouping correlated strongly with MLST ST (Technical Appendix Figure 2). PFGE was more discriminative than MLST because each ST was represented by >1 PFGE pattern.

### MLST Analysis

Irrespective of source, most isolates had a close genetic background (Table 4). Ninety percent of all isolates were either ST21 or ST29, both belonging to ST complex 29 and differing by 1 nt in the *adk* locus. Most isolates from continental Europe were ST21 (74%) and *stx_2_*+ (53%). Considerably fewer isolates from humans in Scotland were ST21 (36%) and *stx_2_*+ (18%). Other differences between Scotland and continental Europe were related to the relative absence of *tccP2* in isolates from Scotland and the presence of different alleles upstream of LEE (Table 4). Isolates from cattle were genotypically more similar to those from humans from Scotland than from continental Europe (Table 4), except for the proportion of isolates that were ST21 and *stx*_2_+. This similarity may be explained by the fact that the isolates analyzed from cattle (33 [13%] of 249) were not a random sample of all isolates from cattle but were selected on their virulence properties (i.e., *stx*+ and *eae*+) to assess their potential to cause human infection rather than to provide a direct comparison with isolates from humans in Scotland.

**Table 4:** Results of genotypic characterization by MLST and the presence of virulence genes for *Escherichia coli* O26 isolates, Scotland*

Genotypic characterization	No. (%) Isolates from humans, n = 30		No. (%) isolates from cattle, Scotland, n = 33
	Scotland	Germany/Italy	
MLST			
ST			
21	4 (36.4)	14 (73.7)	22 (66.6)
29	4 (36.4)	5 (26.3)	9 (27.3)
Other	3 (27.2)	0	2 (6.1)
ST complex†			
29	10 (90.9)	19 (100.0)	31 (93.9)
10	1 (9.1)	0	2 (6.1)
*espA*‡			
Allele 1	9 (81.8)	19 (100.0)	31 (100.0)
Other	1 (9.1)	0	0
ND	1 (9.1)	0	0
Upstream of *LEE1*§			
Allele1	2 (18.2)	14 (73.7)	7 (21.2)
Allele2	5 (45.4)	5 (26.3)	20 (60.6)
allele3	0	0	1 (3.0)
allele4	1 (9.1)	0	3 (9.1)
allele5	1 (9.1)	0	0
allele6	1 (9.1)	0	0
ND	1 (9.1)	0	2 (6.1)
Presence of virulence genes			
* stx*			
*stx*–	6 (54.5)	4 (21.1)	7 (21.2)
*stx*_1_ +	3 (27.3)	5 (26.3)	16 (48.5)
*stx_2_*+	2 (18.2)	10 (52.6)	10 (30.3)
*Eae*¶			
Absent	1 (9.1)	0	2 (6.1)
Present	10 (90.9)	19 (100.0)	31 (93.9)
*sepL*#			
Absent	1 (9.1)	0	2 (6.1)
Present	10 (90.9)	19 (100.0)	31 (93.9)
hlyA**			
Absent	3 (27.3)	7 (36.8)	6 (18.2)
Present	8 (72.7)	12 (63.2)	27 (81.8)
*tccP*††			
Absent	11 (100.0)	19 (100.0)	33 (100.0)
Present	0	0	0
*tccP2*††			
Absent	6 (54.5)	2 (10.5)	16 (48.5)
Present	5 (45.5)	17 (89.5)	17 (51.5)

*MLST, multilocus sequence typing; ST, sequence type; ND, not determined; stx, Shiga toxin; +, *stx* gene present; –, *stx* gene absent. *LEE1* encodes the first operon of the locus of enterocyte effacement (LEE) and the 644-bp region sequenced includes the promoter for this operon amplified using the defined LEE primer pair in Table 2. †The ST and ST complex were assigned in accordance with the *E. coli* MLST database (http://mlst.ucc.ie/mlst/dbs/Ecoli). ‡*espA* encodes for the surface-associated protein, espA. The allele numbers at each loci were assigned in the order in which they were discovered. §Six different sequences were discovered for the region upstream of *LEE1* in the *E. coli* O26 isolates. Approximately 644 bp of sequence data were determined, and all sequence variation between the *E. coli* O26 alleles occurs within a 91-bp region upstream of *LEE1* in the region where regulators act in *E. coli* O157:H7. The allele numbers at each locus were assigned in the order in which they were discovered. ¶*eae*, gene that encodes intimin. *#sepL*, gene confirming the presence of LEE pathogenicity island. **hlyA, enterohemolysin. ††*tccP/tccP2,* genes encoding Tir-cytoskeleton coupling protein and Tir-cytoskeleton coupling protein 2, which are used in actin polymerization and subsequent attaching and effacing lesion formation.

### Disease Severity Association

Before NMS analysis, 3 isolates were excluded because of missing data. ST complex, LEE, *tccP*, and *eae* were removed because no variation existed in the data (Table 4). The final matrix comprised 5 variables and 27 isolates. The final NMS solution was 3-dimensional and explained 90.8% of the variation in clinical severity and explained more variation than expected by chance (Monte Carlo, p = 0.008). Final stress for the 3-dimensional solution was 8.53 with no real risk for drawing false associations (*32*), and the final instability was 0 with 53 iterations.

**Figure 3:**
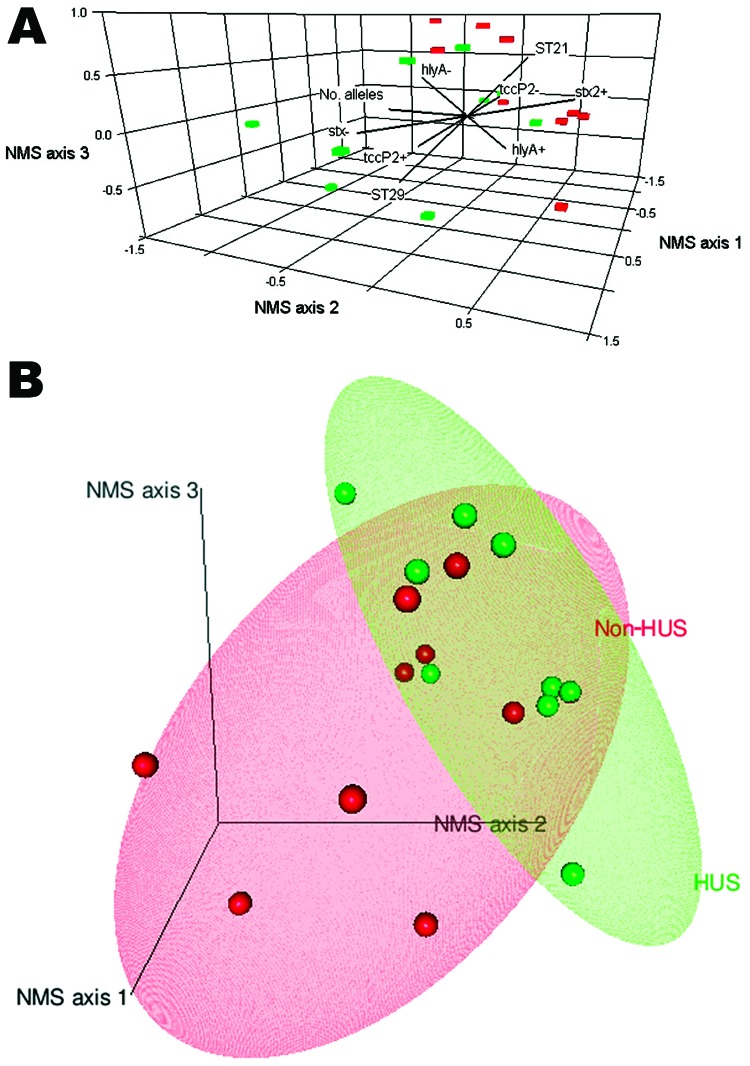
Patterns in data associated with the clinical severity of *Escherichia coli* O26 infection in humans, as identified by nonmetric multidimensional scaling, Scotland. A) Joint graph illustrating the association between the multilocus sequence typing and genotypic variables measured and the severity of the human infection (hemolytic uremic syndrome [HUS] in red and non-HUS [diarrhea, bloody diarrhea] in green). B) 3-dimensional scatterplot and 80% confidence ellipses (R rgl package [*33*]) around the cases in space illustrating the separation between individual classes as HUS (green) and non-HUS (red).

The results of the NMS models are shown in joint graphs (Figure 3, panel A) and 3-dimensional ordination graphs (Figure 3, panel B) of the distance between sample units, which approximates dissimilarity in clinical severity. Where appropriate, R package version 2.12.1 (*33*) was used to add 80% confidence ellipsoids to discriminate between groups of interest. Variables used in the NMS analysis are shown as vectors; the direction indicates positive and negative correlation (Figure 3, panel A). Although some overlap exists, the difference between HUS and non-HUS groups was statistically significant (multiresponse permutation procedures, p = 0.007) (Figure 3, panel B). HUS was associated with ST21 and *stx_2_*, whereas non-HUS cases were associated with ST29 and the absence of *stx_2_*. The *hlyA* gene was not associated with either condition because 69% of non-HUS isolates possess *hlyA* and 67% of HUS isolates possess *hlyA* (Figure 3, panel A).

## Discussion

Although Stx-producing *E. coli* O26 is a major cause of HUS in continental Europe, human infection with this pathogen is uncommon in Scotland and has resulted in less severe illness. In contrast, *E. coli* O157 is a major cause of human infections and HUS (*1*). Previous reports of a high prevalence of *E. coli* O26 in cattle (*21*) do not appear to be matched by a high level of infection in humans. Our first objective was to determine whether this discrepancy could be explained by the natural heterogeneity in strains by examining virulence determinants associated with serious human disease in strains recovered from cattle in Scotland.

**Figure 4:**
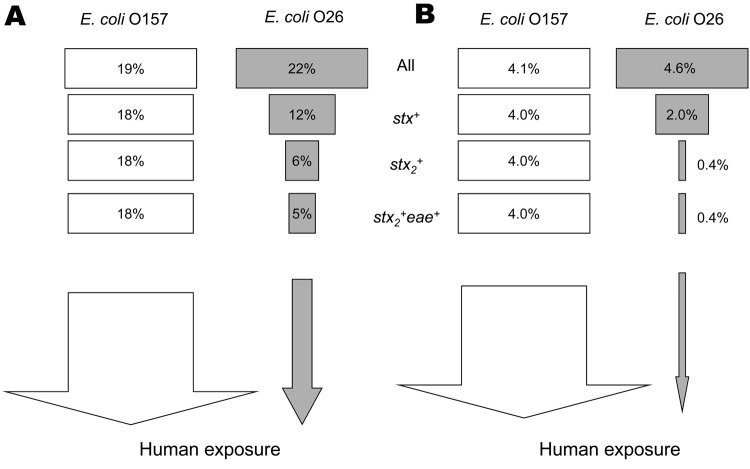
Schematic comparing farm-level (A) and animal-level (B) prevalences of *Escherichia coli* O157 and *E. coli* O26 in Scotland for different virulence levels. Analysis was done by using only the 338 farms sampled for both *E. coli* O157 and *E. coli* O26.

The overall percentage of farms on which *E. coli* shedding was detected and the overall proportion of fecal pats positive were comparable for both organisms, but for *E. coli* O26, the farm and pat-level prevalence becomes smaller as the carriage of the virulence determinants *eae* and *stx* are taken into account (Figure 4). In contrast, the prevalence of virulent strains of *E. coli* O157 (*stx*+*eae*+) is not smaller. Thus, because there are fewer virulent strains at farm and animal levels, the risk for human illness is expected to be much lower for *E. coli* O26 than for *E. coli* O157 in Scotland.

Our second objective was to determine the genetic relationship of strains from different sources. PFGE characterization demonstrated that PFGE profiles of isolates from humans differed from those from cattle. This result was expected in the absence of a demonstrable epidemiologic link. However, human strains are widely distributed among groupings of isolates from cattle. Although an exact interpretation of relatedness of isolates from humans to animals is not possible, the isolates cannot be regarded as distinct populations. Further characterization by MLST confirmed that the genetic backbone of bovine and human *E. coli* O26 strains in Scotland match and that isolates from cattle in Scotland are closely related to highly pathogenic isolates from humans in Germany and Italy.

Characterization of human strains from Scotland and continental Europe and of bovine strains from Scotland showed that most *E. coli* O26 strains were ST29 and were likely to be capable of expressing a T3S system because they were *eae* and *sepL* positive. Although less virulent strains generally are present in cattle in Scotland, the unexpected finding of this study was that the genetic background of many of the strains shows a close relationship to strains causing severe human infections in continental Europe.

The use of NMS enables identification of an association between HUS and ST21 strains and confirms the role of *stx_2_* alone in severe human disease. As previously suggested, the *hlyA* gene was not associated with HUS (*8*). Our results indicate that, in addition to *stx_2_*, *tccP2* is linked to the severity of clinical disease. Therefore, the risk to humans in Scotland from isolates from cattle might be even lower for *E. coli* O26 if ST21 and the carriage of *tccP2* are considered.

In the absence of *stx* genes, isolates from cattle feces are less of a threat to human health than *E. coli* O157 strains, but there are *E. coli* O26 in Scotland of ST21 that are LEE positive and carry *tccP2*. These need only to acquire the *stx_2_*-encoding bacteriophage and they could prove to be highly pathogenic because *E. coli* O26 is a highly dynamic group capable of acquiring *stx*-encoding phages (*34**,**35*). Zhang et al. (*14*) described a shift in *stx* genotype from *stx_1_* to *stx_2_*: before 1994, only the *stx_1_* gene was identified in their STEC O26 isolate collection, but after 1997, *stx_2_* was the only genotype identified in 71% of the isolates. The trigger for this shift was not established.

At the time of our study, there was no correlation between farms in Scotland with *E. coli* O157 and *E. coli* O26 (p = 0.28) or those with *E. coli* O26 *stx_1_*+ *stx_2_*+ and *E. coli* O157 (p = 0.10). However, release of free *stx*-encoding bacteriophages into the intestinal environment (*36**,**37*) by STEC O157 or other EHEC may permit horizontal transfer of virulence genes to *E. coli* O26. Acquisition of *stx_2_*-encoding bacteriophages could confer *E. coli* O26 with greater pathogenic potential for humans, and possession of *stx_2_* may alter the bovine immune response and lead to longer shedding and to larger quantities of the shed bacterium, resulting in greater exposure to humans. Changes in virulence characteristics of strains may also affect pathogen transmission in cattle (*38*) and thus pose a greater threat to humans from an increase in bacterial prevalence.

We suggest that limited human exposure in Scotland to *E. coli* O26 with virulence genes explains the recorded difference in the number of human infections attributed to *E. coli* O26 or *E. coli* O157. In addition, the more virulent forms of *E. coli* O26 (*stx_2_*+*eae*+) in cattle in Scotland were clustered in the sparsely populated northeast region. However, exposure routes and exposure doses for *E. coli* O26 and serogroup O157 may differ, although no evidence confirms this alternative explanation for the human infection rate differences. Although cattle are hypothesized as a potential reservoir of *E. coli* O26 (*18*), little is known about the transmission routes to humans and the only evidence linking human infection with cattle is 2 HUS cases in Austria indirectly linked to consumption of unpasteurized milk (*20*).

**Figure 5:**
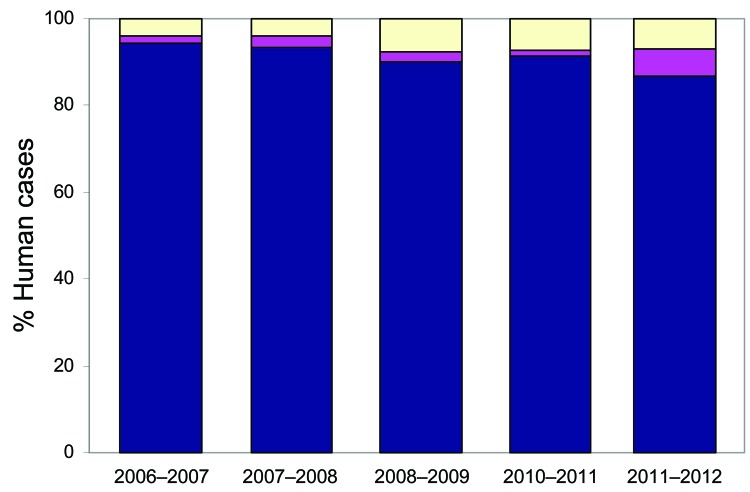
*Escherichia coli* O157 and Shiga toxin–producing non-O157 *E. coli* infection in humans in Scotland identified or confirmed by the Scottish *E. coli* O157/VTEC Reference Laboratory, Edinburgh, UK, by financial year (April–March). Samples include isolates, feces, and serum. Non-O157 isolates were serotyped by the Laboratory of Gastrointestinal Pathogens. Data are presented as percentage of all cases in humans that are *E. coli* O157 (blue), Shiga toxin-producing non-O157 (excluding *E. coli* O26) (yellow) and Shiga toxin–producing *E. coli* O26 (pink). The mean number of cases per financial year during 2006–2011 was 248 (221–275). This time frame was selected to ensure application of consistent method.

The approach to laboratory diagnosis of non-O157 *E. coli* gastrointestinal infection in humans has changed in recent years. At the time of this study, testing of samples from humans for non-O157 *E. coli* was conducted by a small number of clinical diagnostic laboratories by using polyvalent antiserum. *E. coli* O26 isolates were forwarded to the SERL for virulence typing by PCR. SERL identified additional *E. coli* O26 isolates by testing feces from patients with suspected STEC infection but who had negative routine culture results. However, since 2006–2007, non-O157 STEC detection in submitted fecal samples has been conducted solely by SERL by using PCR followed by culture and characterization of STEC. Since 2006–2007, the reported incidence of *E. coli* O26 in Scotland has increased from 1.6% of all STEC identified at SERL to 6% in 2010–2011 (Figure 5). The virulence patterns of *E. coli* O26 strains isolated from humans in Scotland also appear to have altered. The *stx_1_*+ *E. coli* O26 isolates previously predominated, but in 2010–2011, >50% of *E. coli* O26 isolates possessed both *stx*_1_ and *stx_2_*, and for the first time, 1 isolate from human feces was solely positive for *stx*_2_.

In 2010, three particularly severe sporadic cases of HUS associated with *E. coli* O26 in children were reported in Scotland (*39*). The characteristics of 2 of the *E. coli* O26 strains included *stx_1_* and *stx_2_* (*39*). To our knowledge, this was the first time >1 *E. coli* O26–attributed HUS case was reported in Scotland in a calendar year, reinforcing Bettelheim’s (*40*) warning that we ignore these strains at our peril. Worldwide, reports of human outbreaks of non-O157 EHEC, including *E. coli* O26, are common (*2*). In addition, the recent outbreak *of E. coli* O104:H4 in Germany highlights the potential for Shiga toxin prophage acquisition into different *E. coli* genetic backgrounds with serious consequences for human health (*3*).

## Supplementary Material

Technical Appendix Analysis of Shiga toxin–producing *Escherichia coli* O26 isolates in cattle and humans from Scotland, England, Ireland, Belgium, Sweden, and Italy.
