# Supplementary material for: Pathogenic Potential to Humans of Bovine Escherichia coli O26, Scotland
Source: Emerg Infect Dis. 2012 Mar;18(3):439–48. doi: 10.3201/eid1803.111236 (PMC3309639; doi:10.3201/eid1803.111236)
Supplement: Technical Appendix — Analysis of Shiga toxin–producing Escherichia coli O26 isolates in cattle and humans from Scotland, England, Ireland, Belgium, Sweden, and Italy. [file 11-1236-Techapp_2p.pdf]

Article DOI: http://dx.doi.org/10.3201/eid1803.111236

Pathogenic Potential to Humans of Bovine *Escherichia coli* O26, Scotland

Technical Appendix

Analysis of Shiga toxin–producing *Escherichia coli* O26 isolates in cattle and humans from Scotland, England, Ireland, Belgium, Sweden, and Italy.

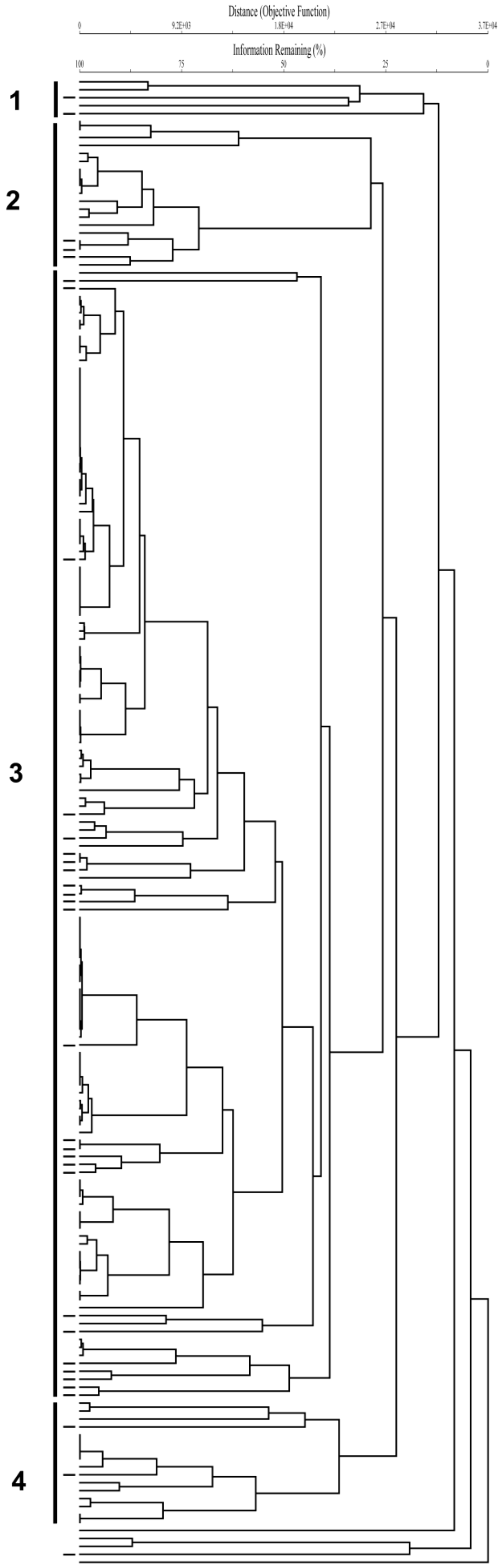

Technical Appendix Figure 1. Dendrogram of 152 cattle and 33 humans with Shiga toxin–producing Escherichia coli O26 isolates from Scotland, England, Ireland, Belgium, Sweden, and Italy (Table 1). Horizontal line denotes the position of isolates from humans. Scale bar indicates degree of similarity (%).

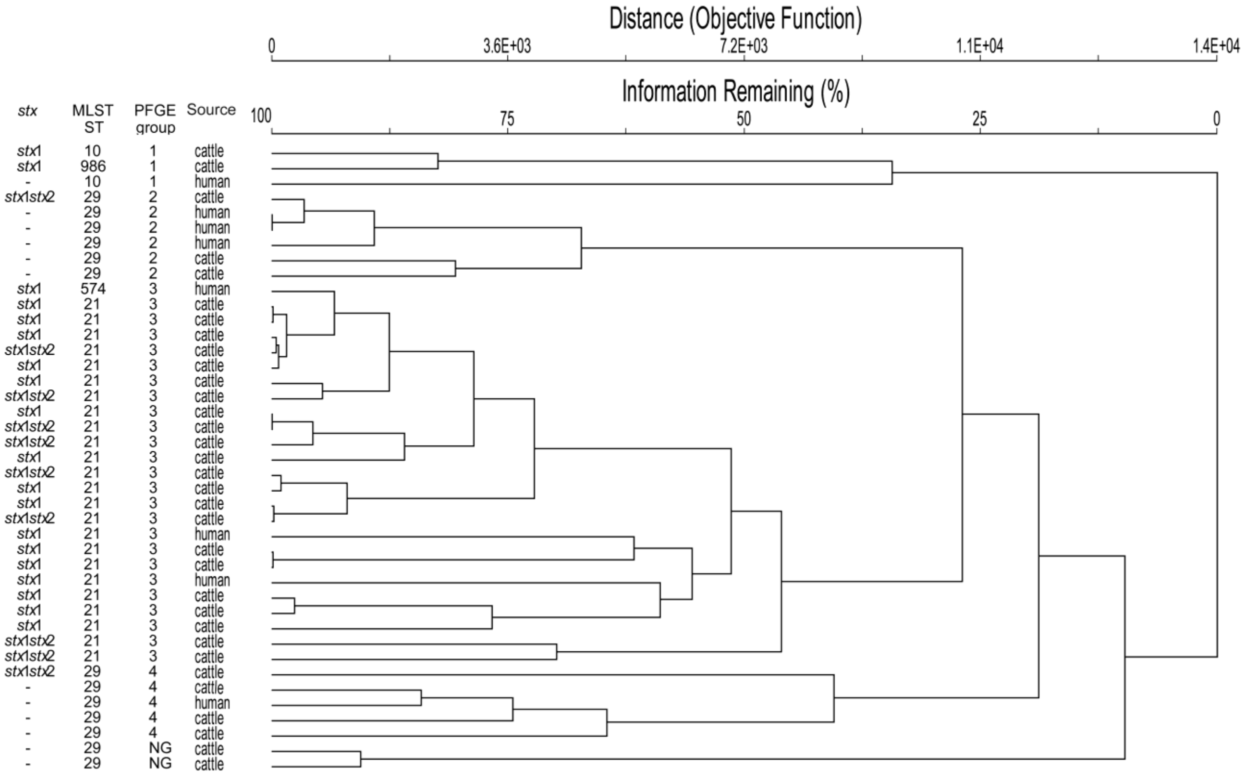

Technical Appendix Figure 2. Shiga toxin-producing Escherichia coli O26 isolated from cattle and humans in Scotland during 2002–2004. stx, Shiga toxin gene; MLST, multilocus sequence typing. Scale bar indicates degree of similarity (%). NG, no group assigned.
